# Author Correction: Estimation of R0 for the spread of SARS-CoV-2 in Germany from excess mortality

**DOI:** 10.1038/s41598-022-26627-8

**Published:** 2022-12-21

**Authors:** Juan Pablo Prada, Luca Estelle Maag, Laura Siegmund, Elena Bencurova, Chunguang Liang, Eleni Koutsilieri, Thomas Dandekar, Carsten Scheller

**Affiliations:** 1 Department of Bioinformatics, Biocenter, Am Hubland, University of Würzburg, 97074 Würzburg, Germany; 2 Institute of Virology and Immunobiology, University of Würzburg, Versbacher Str. 7, 97078 Würzburg, Germany

Correction to: *Scientific Reports* 10.1038/s41598-022-22101-7, published online 14 October 2022

The original version of this Article contained an error in the spelling of the author Chunguang Liang, which was incorrectly given as Liang Chunguang.

The original Article has been corrected.

